# Plant MYB Transcription Factors: Their Role in Drought Response Mechanisms

**DOI:** 10.3390/ijms160715811

**Published:** 2015-07-13

**Authors:** Elena Baldoni, Annamaria Genga, Eleonora Cominelli

**Affiliations:** 1 Dipartimento di Scienze Agrarie e Ambientali—Produzione, Territorio, Agroenergia, Università degli Studi di Milano, Via Celoria 2, 20133 Milano, Italy; E-Mail: elena.baldoni@unimi.it; 2 Istituto di Biologia e Biotecnologia Agraria, CNR, Via Bassini 15, 20133 Milano, Italy; E-Mail: genga@ibba.cnr.it

**Keywords:** MYB, drought, stress response, crosstalk

## Abstract

Water scarcity is one of the major causes of poor plant performance and limited crop yields worldwide and it is the single most common cause of severe food shortage in developing countries. Several molecular networks involved in stress perception, signal transduction and stress responses in plants have been elucidated so far. Transcription factors are major players in water stress signaling. In recent years, different MYB transcription factors, mainly in *Arabidopsis thaliana* (L.) Heynh. but also in some crops, have been characterized for their involvement in drought response. For some of them there is evidence supporting a specific role in response to water stress, such as the regulation of stomatal movement, the control of suberin and cuticular waxes synthesis and the regulation of flower development. Moreover, some of these genes have also been characterized for their involvement in other abiotic or biotic stresses, an important feature considering that in nature, plants are often simultaneously subjected to multiple rather than single environmental perturbations. This review summarizes recent studies highlighting the role of the MYB family of transcription factors in the adaptive responses to drought stress. The practical application value of MYBs in crop improvement, such as stress tolerance engineering, is also discussed.

## 1. Introduction

As the world population is expected to reach nine billion by 2050, crop yields need to be improved by 40% in areas where drought is likely to occur by 2025 [1]. Moreover, frequent occurrences of drought and abnormal weather events have lately been observed all over the world. A study on the effects of all major US weather disasters with costs that exceeded a billion dollars each, between 1980 and 2012, indicates that drought alone caused $50 billion worth of damage to agricultural production [2]. Moreover, current climate prediction models indicate a gradual increase in temperature with a consequent enhancement in the frequency and amplitude of heat and drought stress in the near future that could drastically impact crop production worldwide [3,4].

One of the strategies to overcome these problems consists in the generation of crops with increased tolerance to drought, through biotechnological approaches and advanced molecular breeding techniques. The prerequisite for this strategy is the identification of traits of interest and then of the genes responsible for the determination of these traits. Several efforts have been made to identify candidate genes and to manipulate them in order to obtain drought tolerant plants [5]. Transcription factors (TFs) are key players in the regulatory networks underlying plant responses to abiotic stresses [6]. Among them, different *MYB* genes have been studied for their involvement in the regulation of abiotic stress response, as recently reviewed [7]. Here, we will focus on *MYB* genes functionally characterized for their involvement in drought stress response and eventually in other abiotic or biotic stresses, as summarized in Table 1.

MYB TFs are characterized by the presence of the MYB domain involved in DNA binding. The classification criterion used for MYB proteins is based on the number of repeats present in their sequences, varying from one to four. Each repeat consists of about 52 amino acid residues and forms three α-helices, the second and the third ones are involved in the formation of a helix–turn–helix (HTH) fold [8]. The prototypic mammalian c-Myb protein consists of three repeats in its MYB domain, called R1, R2 and R3, and belongs to the 3R-MYB class. This group of proteins is mainly involved in the regulation of the cell cycle in different organisms, plants included [8].

In plants, the majority of MYB proteins belong to the R2R3-MYB subfamily, whose members contain two repeats. More than 100 members of this class have been described in different species. Based on the conservation of the DNA binding domain and of amino acid motifs in the C terminal domains, the members of this subfamily have been classified and divided into 23 subgroups [8]. The R2R3-MYB TFs play central roles in the control of plant-specific processes, including primary and secondary metabolism, cell fate and identity, development, response to abiotic and biotic stresses [8]. In plants, the 3R-MYB class represents a very small group with only five members [8]. Interestingly, two 3R-MYB proteins, OsMYB3R-2 from rice (*Oryza sativa* L.) and TaMYB3R1 from wheat (*Triticum aestivum* L.), as well as having the more traditional role of 3R-MYB proteins in cell cycle regulation, are also involved in the regulation of drought response and will be described in this review [9,10]. MYB TFs with four R1/R2-like repeats are very rare; only one member has been described in different plant species and none is involved in the drought response [8]. MYB TFs with a single or a partial repeat, collectively called “MYB-related”, are grouped in different subclasses, depending on the presence of particular repeats [8]. They are involved in different processes, such as the control of cellular and organ morphogenesis, secondary metabolism and circadian rhythm. Among them, a role in drought response, and in particular in the regulation of stomatal movements, has been described only for the StMYB1R-1 TF from potato (*Solanum tuberosum* L.), as reported below [11].

**Table 1:** *MYB* genes involved in drought response. The different functional analyses on *MYB* genes were performed on knockout mutants (KO), or on overexpression (OE), virus-induced gene silencing (VIGS) and RNA-interference (RNAi) lines.

Gene Name	Locus/Accession Nr	Species	Transgenic Plants/Mutants	References
**Root**				
*AtMYB77*	AT3G50060	*Arabidopsis thaliana*	*Arabidopsis* KO	[12,13]
*AtMYB60*	AT1G08810	*Arabidopsis thaliana*	*Arabidopsis* OE	[14]
*AtMYB96*	AT5G62470	*Arabidopsis thaliana*	*Arabidopsis* activation tagged OE	[15]
*MdSIMYB1*	KC691248	*Malus x domestica*	apple OE, tobacco OE	[16]
**Leaf**				
*NbPHAN*	FR878011	*Nicotiana benthamiana*	*N. benthamiana* VIGS	[17]
**Stomata**				
*AtMYB88*	AT2G02820	*Arabidopsis thaliana*	*Arabidopsis* KO	[18,19,20,21,22]
*FLP/AtMYB124*	AT1G14350	*Arabidopsis thaliana*	*Arabidopsis* KO	[18,19,20,21,22]
*AtMYB60*	AT1G08810	*Arabidopsis thaliana*	*Arabidopsis* OE, KO	[14,23]
*VvMYB60*	ACF21938	*Vitis vinifera*	complementation of *Arabidopsis* mutant	[24]
*AtMYB96*	AT5G62470	*Arabidopsis thaliana*	*Arabidopsis* activation tagged OE, KO	[15,25]
*AtMYB20*	AT1G66230	*Arabidopsis thaliana*	*Arabidopsis* OE, KO	[26]
*AtMYB61*	AT1G09540	*Arabidopsis thaliana*	*Arabidopsis* OE, KO	[27,28,29,30]
*AtMYB15*	AT3G23250	*Arabidopsis thaliana*	*Arabidopsis* OE	[31]
*AtMYB44*	AT5G67300	*Arabidopsis thaliana*	*Arabidopsis* OE	[32]
*GbMYB5*	JF820389	*Gossypium barbadense*	cotton VIGS, tobacco OE	[33]
*StMYB1R-1*	AU279205	*Solanum tuberosum*	Potato OE	[11]
*TaMYB3R1*	HQ236494	*Triticum aestivum*	*Arabidopsis* OE	[10]
**Flower**				
*AtMYB21*	AT3G27810	*Arabidopsis thaliana*	*Arabidopsis* KO	[34,35,36]
*CmMYB2*	JF795918	*Chrysanthemum morifolium*	*Arabidopsis* OE	[37]
**Cell Wall**				
*AtMYB52*	AT1G17950	*Arabidopsis thaliana*	*Arabidopsis* OE, activation tagged OE	[38]
**Cuticle**				
*AtMYB94*	AT3G47600	*Arabidopsis thaliana*	*Arabidopsis* OE	[39]
*AtMYB96*	AT5G62470	*Arabidopsis thaliana*	*Arabidopsis* OE; Camelina OE	[15,40,41]
*EsWAX1*	BAJ34253	*Eutrema salsugineum*	*Arabidopsis* OE	[42]
**Suberin**		**		
*AtMYB41*	AT4G28110	*Arabidopsis thaliana*	*Arabidopsis* OE	[43,44,45,46]
**Flavonoids**				
*AtMYB12/PFG1*	AT2G47460	*Arabidopsis thaliana*	*Arabidopsis* OE	[47]
*AtMYB75/PAP1*	AT1G56650	*Arabidopsis thaliana*	*Arabidopsis* OE	[47]
*MdMYB10*	ABB84753	*Malus x domestica*	*Arabidopsis* OE	[48,49]
**Abiotic Stresses**				
*AtMYB2*	AT2G47190	*Arabidopsis thaliana*	*Arabidopsis* OE, KO	[50,51,52,53]
*AtMYB20*	AT1G66230	*Arabidopsis thaliana*	*Arabidopsis* OE, KO	[26,54]
*OsMYB3R-2*	LOC_Os01g62410/ Os01g0841500	*Oryza sativa*	*Arabidopsis* OE	[9,55]
*OsMYB2*	LOC_Os03g20090/ Os03g0315400	*Oryza sativa*	Rice OE, RNAi	[56]
*OsMYB48-1*	LOC_Os01g74410/ Os01g0975300	*Oryza sativa*	Rice OE	[57,58]
*TaMYB2A*	AY615199	*Triticum aestivum*	*Arabidopsis* OE	[59]
*TaMYB19*	JF951903	*Triticum aestivum*	*Arabidopsis* OE	[60]
*TaMYB30-B*	JF951913	*Triticum aestivum*	*Arabidopsis* OE	[61]
*TaMYB33*	JN584645	*Triticum aestivum*	*Arabidopsis* OE	[62]
*GmMYBJ1*	KC751453	*Glycine max*	*Arabidopsis* OE	[63]
*CpMYB10*	AF510112	*Craterostigma plantagineum*	*Arabidopsis* OE	[64]
*MdoMYB121*	KC834015	*Malus x domestica*	apple OE; tomato OE	[65]
*MdSIMYB1*	KC691248	*Malus x domestica*	apple OE, tobacco OE	[16]
*PtsrMYB*	–	*Poncirus trifoliata*	tobacco OE	[66]
**Abiotic and Biotic Stresses**				
*AtMYB12/PFG1*	AT2G47460	*Arabidopsis thaliana*	tobacco OE	[67,68,69]
*AtMYB75/PAP1*	AT1G56650	*Arabidopsis thaliana*	tobacco OE	[68]
*AtMYB44*	AT5G67300	*Arabidopsis thaliana*	*Arabidopsis* OE, KO; soybean OE	[32,70,71,72,73]
*AtMYB96*	AT5G62470	*Arabidopsis thaliana*	*Arabidopsis* activation tagged OE	[74,75]
*AtBOS1/MYB108*	AT3G06490	*Arabidopsis thaliana*	*Arabidopsis* KO	[76]
*AtMYB15*	AT3G23250	*Arabidopsis thaliana*	*Arabidopsis* OE, KO	[77,78,79,80,81]
*OsMYB4*	LOC_Os04g43680/ Os04g0517100	*Oryza sativa*	OE in rice, *Arabidopsis*, apple, tomato, potato, sage, tobacco, *Osteospermum*, barley	[82,83,84,85,86,87,88,89,90,91,92]
*TaPIMP1*	CN011324	*Triticum aestivum*	tobacco OE; wheat OE, RNAi	[93,94]
*SpMYB*	–	*Solanum pimpinellifolium*	tobacco OE	[95]
**Other**				
*QsMYB1*	JF970262 and JF970262	*Quercus suber*	–	[96,97]
*AtMYB33*	AT5G06100	*Arabidopsis thaliana*	–	[98,99,100,101]
*AtMYB101*	AT2G32460	*Arabidopsis thaliana*	–	[98,99,100,101]
*StGAMyb-like1*	–	*Solanum tuberosum*	–	[102]
*StGAMyb-like2.1*	–	*Solanum tuberosum*	–	[102]
*StGAMyb-like2.2*	–	*Solanum tuberosum*	–	[102]
*TaMYB2*	AB252145	*Triticum aestivum*	–	[103]
*QsMYB1*	JF970262 and JF970262	*Quercus suber*	–	[96,97]

With the exception of OsMYB3R-2, TaMYB3R1 and StMYB1R-1, all the other *MYB* genes described in this review code for R2R3-MYB proteins, since this is the class of plant MYB TFs primarily involved in the response to environmental stresses.

The expression of many *MYB* genes is regulated by drought. For example, in rice it was reported that 65% of *MYB* genes expressed in seedlings were differentially regulated under drought stress [104]. An even higher percentage is observed in *Arabidopsis thaliana* (L.) Heynh.: transcriptomic data collected in the GENEVESTIGATOR database [105] show that 51% of *AtMYB* genes are up-regulated by drought and 41% are down-regulated [104]. Besides transcriptomic data, different MYB TFs have been functionally characterized for their involvement in one or more mechanisms of drought response, as summarized in Table 1. In recent years, it has become clear that the traditional classification of MYB function (regulation of development, defense, metabolism) cannot be very rigid. For instance, some MYB TFs, previously characterized for their role in the regulation of a specific developmental process, have later been described for their response to specific stresses or *vice versa*, as shown in Table 1 and extensively discussed in this review.

The goal of this review is to focus only on *MYB* genes functionally characterized for their involvement in drought response, through analysis of mutants, silenced and overexpressing lines (Table 1). Although expression data from many other *MYB* genes suggest a possible role in drought response, these genes have not been included in this review, since the available data were not thorough enough to allow us to speculate about the physiological processes in which they may be involved.

**Figure 1:**
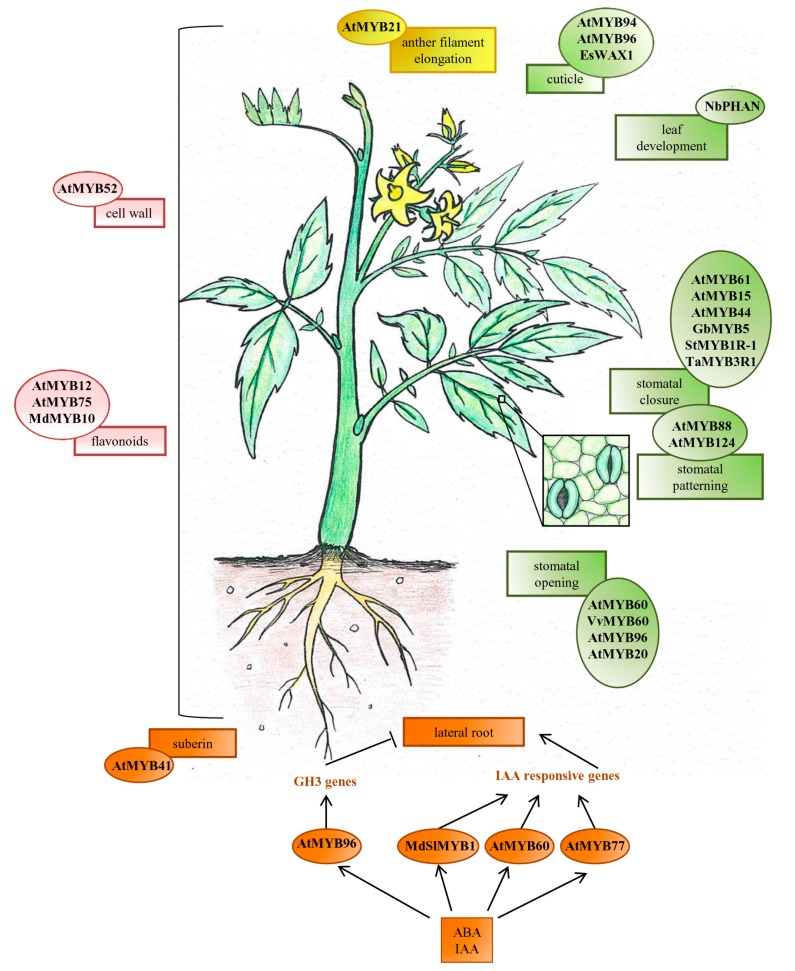
Network of R2R3-MYB transcription factors well-characterized for their regulatory roles in different organs, specific cell-types and metabolic pathways in response to drought stress. Line arrows represent a positive regulation, while line ending with a bar indicates a negative regulation. Processes and pathways related to vegetative organs, flower, root or whole plant are colored in green, yellow, orange and pink, respectively.

The genes described here are classified depending on the process(es) in which they are involved. In some cases, the same gene has been described for its involvement in different mechanisms of drought response, and in these cases all its functions have been reported. The classification addressed here includes the following processes regulated by MYB TFs: development, growth and function of organs and specific cell-types (root, leaf, stomata, flower) and metabolite biosynthesis (cell wall components, cuticle, suberin and flavonoids), as summarized in Figure 1.

Moreover, it is well known that plants are often exposed to different stresses simultaneously, and the interaction of drought stress with other environmental cues has deleterious effects on plant growth [106]. Hence, we have dedicated the section “Crosstalk among different stress responses” to *MYB* genes described for their involvement in other stresses besides drought. Finally, the last section is dedicated to the post-transcriptional control of some *MYB* genes involved in drought response.

## 2. Development, Growth and Function of Organs and Specific Cell-Types

### 2.1. Root

Root system size, properties and distribution ultimately determine plant access to water. Although roots were traditionally difficult to study, recent progress has made the manipulation of root architecture and physiology a feasible strategy to produce crops with better yields [107]. There is great interest from geneticists and breeders in developing plants with root traits that will be useful to improve productivity under drought. However, a deeper understanding of these traits and their relation to plant strategies to increase crop productivity under drought is needed. Generally, the root traits that contribute to maintaining productivity under water stress, are: small fine root diameter, long specific root length and considerable root length density (root length per soil volume), especially at depths where water is available in the soil [108]. Lateral roots are the most active portion of the root system in water uptake. Lateral root growth is suppressed under osmotic stress during a first quiescent phase in an abscisic acid (ABA)-dependent manner, and, during the recovery phase, it starts to reactivate, through a process in which ABA synergistically acts with auxin to promote lateral root development [12].

Some *MYB* genes are involved in drought response through their regulation of lateral root growth. Interestingly, the AtMYB77 protein, previously described as a positive regulator of lateral root growth through auxin signaling [13], is involved in regulating this recovery phase through the interaction with the PYL8 ABA receptor [12]. Zhao and colleagues showed that in *myb77* mutant seedlings, lateral root growth was more sensitive to inhibition by ABA than that of wild type seedlings. Moreover, in this mutant the exposure to auxin could reverse ABA-induced inhibition of lateral root growth. The PYL8-AtMYB77 interaction enhances AtMYB77 activity to induce the expression of multiple auxin-responsive genes. Therefore, AtMYB77 represents a key protein mediating crosstalk between ABA and auxin signaling in lateral root development in response to drought. Data collected by the same authors support a possible redundant role of AtMYB44 and AtMYB73, belonging to subgroup 22 as AtMYB77 [12].

Two *Arabidopsis* genes, *AtMYB60* and *AtMYB96*, which are well characterized for their role in stomatal movements (see Section 2.3), are also involved in the regulation of lateral root growth. *AtMYB60* expression in roots was induced by auxin, and *Arabidopsis* plants overexpressing this gene, grown on MS plates containing mannitol, developed a greater root mass [14]. The authors proposed a model in which AtMYB60 may induce root growth, increasing the capacity for water uptake during the initial stages of stress.

*AtMYB96* expression was significantly induced by drought and ABA and moderately induced by high salt. In an activation tagging mutant line overexpressing *AtMYB96*, lateral root growth and density were significantly reduced, while in the knockout mutant no difference was observed in comparison with the wild type [15]. Data from Seo and collaborators [15] strongly support the idea that AtMYB96 is a key factor that integrates ABA and auxin signals in modulating auxin homeostasis during lateral root development, particularly under water deficit conditions, through the regulation of a subset of *GH3* genes encoding auxin-conjugating enzymes.

In crops, only one *MYB* gene involved in root growth and in drought response has been described [16]. The expression of the apple (*Malus x domestica* Borkh) *MdSIMYB1* gene is markedly induced by salt, cold and polyethylene glycol (PEG)-mediated osmotic treatments, as well as by an immediate ethylene precursor (1-aminocyclopropane-1-carboxylic acid), and several hormones: ABA, indole-3-acetic acid (IAA), methyl-jasmonate (MeJA) and salicylic acid (SA). The ectopic expression of *MdSIMYB1* in *Nicotiana benthamiana* Domin led to insensitivity of seed germination to ABA and NaCl treatments. Moreover, *MdSIMYB1*-expressing *N. benthamiana* plants displayed an enhanced tolerance to salt, drought and cold stresses. An expression analysis revealed that the transcript level of some stress-responsive genes (*NtDREB1A*, *NtERD10B* and *NtERD10C*) and some auxin-responsive genes (*NtIAA4.2*, *NtIAA4.1* and *NtIAA2.5*), which have been associated with root growth in *N. benthamiana*, was increased in transgenic *N. benthamiana* compared to wild type under standard growth conditions. Interestingly, transgenic plants generated more robust root systems than wild type plants, both under control and stress (salt, drought and cold) conditions. These observations, taken together with the IAA-mediated induction of *MdSIMYB1* expression, suggested that this gene could be involved in auxin response and promote root growth by regulating the expression of auxin-responsive genes [16]. These data have been further confirmed in *MdSIMYB-*overexpressing apple plants, which showed more robust root systems compared to wild type plants, as well as an improved tolerance to salt, drought and cold stresses. Moreover, MdSIMYB1 protein is able to interact with AtGL3, as revealed by bimolecular fluorescence complementation (BiFC) into onion epidermis cells [16]. AtGL3 participates in hair and non-hair formation in the root epidermis in *Arabidopsis* [109]. This finding confirmed the involvement of MdSIMYB1 in root growth and development and suggested that MdSIMYB1 may regulate cell fate in the epidermis by interacting with GL3-like plant proteins [16].

### 2.2. Leaf

In leaves, the morphological and physiological responses to drought stress are fundamental to reduce water loss and improve water use efficiency. It is well-known that drought reduces leaf growth by affecting cell division and expansion. However, current understanding of stress-regulated growth is still very fragmentary, partly because studies combining detailed growth analysis and molecular characterization of growing organs are still relatively scarce [110].

An example of a gene that controls leaf development and also drought tolerance was reported from *N. benthamiana* [17]. NbPHAN belongs to the AS1-RS2-PHAN (ARP) protein complex within the R2R3-type MYB subfamily that have two imperfect MYB repeat units in the MYB domain and an evolutionary conserved role in specification of leaf adaxial identity [8]. The newly emerged young leaves of *N. benthamiana* plants agroinfiltrated with a construct able to silence *NbPHAN*, through the virus-induced gene silencing (VIGS) method, showed altered leaf shapes and ectopic growth on the major veins of leaves, but no alterations in other plant organs. Moreover, these plants exhibited impaired tolerance against drought stress and increased water loss, without showing alteration in stomata density. Silencing of *NbPHAN* led to a very low expression of stress-related genes, normally highly expressed under water deficit conditions, such as genes involved in the biosynthesis of polyamines and in reactive oxygen species detoxification. Furthermore, the expression level of *NbDREB*, but not of *NbAREB*, was decreased in silenced plants compared with not-silenced plants under water deficit, suggesting that NbPHAN plays a role during drought stress through an ABA-independent mechanism [17]. These results provide interesting evidence for a new role for the *ARP* genes in drought response, beside their already extensively described function in leaf development.

### 2.3. Stomata

Stomata are small pores present on the epidermis of green tissues that mediate exchanges between the plant and the atmosphere: CO_2_ enters through stomata as the carbon source for photosynthesis, while water vapor is released by transpiration. Stomata are surrounded by a pair of highly specialized cells, called guard cells, whose changes of turgor pressure control opening and closure of stomata. Compared to membrane transport, gene expression has been traditionally believed to be a late event in guard cell signals that regulate stomatal movements. However, more recent data highlighted the fact that the two processes are much more tightly integrated [111]. Interestingly, the guard cell transcriptome is particularly rich in TF-encoding genes [112]. Among them, some *MYB* genes have been described for their role in the regulation of stomatal movements [113]. Moreover, some *MYB* genes previously described for their role in stomatal patterning have also been demonstrated to regulate stomatal movements in response to drought.

The two paralogous R2R3-type MYB FLP (AtMYB124) and AtMYB88 proteins are required to limit cell divisions in the stomatal lineage: in *flp-1* single mutant and *flp-1 myb88* double mutant, guard mother cells undergo extra divisions resulting in stomatal clusters, with the double mutant displaying more and larger clusters than *flp-1* alone [18,19]. These TFs perform this function by regulating expression of cell cycle genes [19,20]. Although their function in epidermal patterning is more extensively described, FLP and AtMYB88 are also required for abiotic stress tolerance [21]. The *flp-1 myb88* double mutant was more susceptible to both drought and salt stresses. The double mutant lost water significantly faster than wild type. Under normal conditions stomatal aperture assays did not reveal any differences between wild type and double mutant. However, the ABA response of stomata was impaired, as a lower percentage of stomata (30%) closed in response to ABA in the double mutant than in wild type (70%), indicating that FLP/AtMYB88 activity is ABA-dependent [21]. Interestingly, transcriptomic analysis performed on green tissues from the double mutant, grown under standard conditions, indicated that FLP and AtMYB88 negatively control the expression of genes associated with stomatal development, but positively regulate the expression of genes related to stress condition. Unlike the *flp-1 myb88* double mutant, *tmm* (too many mouths), another stomatal patterning mutant [22] did not show any differences in the expression pattern of stress-responsive genes compared to wild type. Therefore, the low expression level of stress-responsive genes in the *flp-1 myb88* double mutant is not just an indirect effect of abnormal stomatal complexes, but highlights a specific role for FLP and AtMYB88. Among genes differentially expressed between wild type and the *flp-1 myb88* double mutant, *AtNAC019* is the only immediate target of FLP/AtMYB88 proteins, as shown by ChIP–chip (chromatin immunoprecipitation experiments followed by hybridization of *Arabidopsis* whole-genome tiling arrays) analysis [21]. AtNAC019 is a TF, belonging to the NAC family, involved in the positive regulation of different abiotic stress-responsive genes [114]. It is likely that FLP/AtMYB88 regulators control the abiotic stress-response pathway at least in part by regulating *AtNAC019* gene expression.

Other MYB TFs were characterized for their role in stomatal movement, without being involved in stomatal development or patterning. Some of these proteins have a positive effect on stomatal opening, while most promote stomatal closure.

Among genes involved in the regulation of stomatal opening, *AtMYB60* is the first to have been characterized [23]. In leaves the *AtMYB60* expression is specifically localized in guard cells, is induced by signals that promote stomatal opening, such as white and blue light, and is repressed by signals that promote stomatal closure such as darkness, desiccation and ABA treatment. Leaves from the *atmyb60-1* knockout mutant displayed a reduction in the light-induced aperture of stomatal pores of approximately 30%, compared to wild type ones. This reduction in stomatal opening helps to limit water loss during drought, thus enhancing plant tolerance [23]. Conversely, in plants overexpressing this gene the opposite phenotype was shown [14]. These data indicate that this TF represents a positive regulator of stomatal opening that needs to be silenced in stress conditions. Microarray expression data showed a differential expression between wild type and *atmyb60-1* in genes involved in the response to abiotic stresses and to pathogens, suggesting that AtMYB60 may have a role to integrate different signals [23]. Furthermore, it was shown that the promoter of *AtMYB60* specifically drives strong *GUS* reporter gene activity in stomata of *Arabidopsis* seedlings and adult plants [115]. This promoter maintains its guard cell specific activity also in other dicots, such as tobacco (*Nicotiana tabacum* L.) and tomato (*Solanum lycopersicum* L.), whereas its activity is completely abolished in rice. These data suggest that at least in dicots there is a conserved mechanism for upstream regulation of AtMYB60 and its orthologs [116].

The grape (*Vitis vinifera* L.) ortholog of *AtMYB60*, the *VvMYB60* gene, is the only one that has been characterized. *Arabidopsis* plants transformed with *VvMYB60* promoter-*GUS* transcriptional fusion constructs showed that the expression of this gene is restricted to stomatal guard cells and is down-regulated in response to ABA. *VvMYB60* expression was high in leaves, seeds and ripened berry skins and was down-regulated in response to ABA and high salinity treatments. Unlike *VvMYB30*, a gene very closely located in the phylogenetic tree, *VvMYB60* was able to complement the loss-of-function *atmyb60-1* mutant, indicating that *VvMYB60* is the only true ortholog of *AtMYB60* in the grape genome [24].

AtMYB96, previously described for its role in lateral root growth (Section 2.1), is also involved in the regulation of stomatal opening [15]. *Arabidopsis* activation-tagged plants overexpressing *AtMYB96* exhibited dwarfed growth with altered leaf morphology. These plants showed enhanced resistance to drought, while the null *myb96-1* mutant was susceptible to drought. Under normal growth conditions the stomatal apertures of the knockout mutant and of the overexpressing lines were not evidently different from those of wild type plants. However, in response to ABA and at a higher extent in response to drought, the stomatal aperture was more reduced in *AtMYB96*-overexpressing leaves than in the wild type leaves, while it decreased to a lesser extent in the *myb96-1* mutant leaves in response to both treatments. Expression data on mutant and overexpressing lines suggested that *AtRD22*, (*RESPONSIVE TO DEHYDRATION22*), a well-known marker of abiotic stress response [25], is a target of AtMYB96. These data show that ABA signals, mediated by AtMYB96, induce plant tolerance to water deficit by reducing stomatal opening. Interestingly, this gene seems to control separate pathways in leaf and root. Regulation of stomatal aperture in leaf is modulated by the AtMYB96/AtRD22-mediated pathway, while regulation of auxin metabolism in root depends on the AtMYB96/GH3 pathway [15].

AtMYB20 is involved in the response to numerous stresses and it will be described in more details in the section concerning the crosstalk among different stresses (Section 4.1). Here, it is noteworthy to say that *Arabidopsis AtMYB20*-overexpressing plants displayed insensitivity to ABA in stomatal closure and consequently increased susceptibility to desiccation, while *AtMYB20* knockout mutation intensified the ABA-promoted stomatal closure, thus conferring increased resistance to desiccation. These data indicate that AtMYB20 is most likely to function as a negative regulator of ABA-mediated stomatal closure [26].

As previously mentioned, the majority of the identified MYB proteins that regulate stomatal movements are involved in controlling stomatal pore closure or in inhibiting its opening, as reported below.

AtMYB61 has a pleiotropic role, influencing lignin deposition [27], mucilage production [28], stomatal aperture [29], xylem and lateral root formation [30]. *AtMYB61* is mainly expressed in guard cells in the darkness, when stomata are closed [29]. The *myb61* loss-of-function mutant presented larger stomatal pores than wild type, while the constitutive expression of *AtMYB61* resulted in enhanced stomatal closure. Infrared thermography revealed that the *myb61* loss-of-function mutant plants were cooler than wild type plants, while the gain-of-function *MYB61OE* were warmer. These findings suggest that constitutive expression of this gene results in more-closed stomata, while loss of AtMYB61 activity results in more-open stomata. The activity of this TF in regulating stomatal closure is apparently ABA-independent, as guard cells from both mutant and overexpressing lines were responsive to increasing ABA concentration. The authors proposed a model in which AtMYB61 has an active role in the dark in the inhibition of stomatal opening [29]. Although AtMYB61 plays a clear role in the regulation of stomatal movements, no data on increased drought-tolerance or sensitivity in overexpressors or mutant lines, respectively, have been reported.

Two other *Arabidopsis* genes, *AtMYB15* and *AtMYB44*, involved in the crosstalk between abiotic and biotic stresses and discussed in more details in the dedicated section (Section 4.2), confer increased stomatal closure with consequent improved drought tolerance when overexpressed in plants [31,32].

*GbMYB5*, isolated from cotton (*Gossypium barbadense* L.), seems to be involved in controlling size and aperture of stomata [33]. Its role in the response to drought stress has been recently characterized in *G. barbadense* by VIGS and in tobacco plants by overexpression. The silencing of *GbMYB5* decreased the proline content and antioxidant enzyme activities and compromised the tolerance of *G. barbadense* plantlets to drought stress. On the other hand, its overexpression in tobacco increased the tolerance to drought stress by decreasing the water loss, elevating the proline content and antioxidant enzyme activities and inducing the expression of stress-responsive genes, including antioxidant enzyme encoding genes (*SOD*, *CAT* and *GST*) and two polyamine biosynthesis genes (*SAMDC* and *ADC1*). A reduced transpiration rate, which may contribute to the reduced water loss and subsequent drought tolerance observed in transgenic tobacco plants, could be achieved by a reduced stomatal aperture, since the stomatal size and the rate of stomatal opening were markedly decreased under drought stress in tobacco *GbMYB5-*overexpressing plants, whereas the stomatal densities were essentially similar between transgenic and wild type plants. The data reported by the authors suggest that *GbMYB5* is possibly involved in ABA-signaling pathways, even if it is not clear whether the reduced stomatal aperture in the *GbMYB5*-overexpressing tobacco plants resulted from increased sensitivity of guard cells to endogenous ABA. Moreover, *GbMYB5* probably also acts in the drought response by regulating the biosynthesis of osmolytes and LEA proteins in order to stabilize plant cells and proteins, and by activating an efficient detoxification system to scavenge ROS [33].

As previously mentioned, the only MYB-related TF for which a role in drought response has been described is the potato StMYB1R-1 factor. Potato plants overexpressing *StMYB1R-1* exhibited improved tolerance to drought, with no negative pleiotropic effects on plant performance. These plants closed stomata more rapidly in response to ABA and exhibited reduced rates of water loss. Expression of some stress-related genes was up-regulated in these transgenic plants under normal growth conditions [11].

TaMYB3R1, a wheat 3R-MYB protein, has been described for its involvement in the regulation of stomatal movement [10]. *TaMYB3R1-*expressing *Arabidopsis* plants produced more rosette leaves at the vegetative growth stage, whereas at the reproductive stage they produced more inflorescences. Under standard growth conditions they showed reduction in stomatal aperture compared to wild type. These transgenic lines were more tolerant to drought and salt stress and they increased expression of ABA-dependent and ABA-independent responsive genes compared to the control plants in response to drought, suggesting that TaMYB3R1 affects both pathways [10]. Interestingly, TaMYB3R1 has MSA (Mitosis Specific Activator)-element binding properties, a typical feature of 3R-MYB proteins, with a conserved role in controlling cell cycle processes in animals and plants [117]. Probably, the altered plant development when the gene is overexpressed correlates with its function in regulating cell cycle. However, further investigations are needed for a better comprehension of its role as a cell cycle regulator and as a modulator of stomatal movements.

These results on StMYBR1 and TaMYB3R1 shed light on novel functions of plant MYB-related and 3R-MYB proteins, not previously described for their involvement in the response to abiotic stresses.

### 2.4. Flower

Compared to the numerous studies performed on effects of environmental changes during plant vegetative growth, especially during seed germination and seedling development, few studies have been reported on the effects of abiotic stresses during reproductive growth. Crops such as wheat and rice show partial male sterility under water deficit, leading to significant reductions in grain production [118,119,120]. *Arabidopsis* exhibits female reproductive organ abortion because of salt stress [121,122]. Recently, a transcriptomic analysis has been conducted to characterize expression changes occurring in unopened floral buds after drought stress in *Arabidopsis* [34]. Several genes, which were differentially expressed between control and drought-treated plants, are involved in stress response and reproductive development. Among them, *AtMYB21* expression is induced in the flower by drought. AtMYB21, together with AtMYB24 and AtMYB108, are positive regulators of filament elongation and anther dehiscence via the JA and gibberellic acid (GA) signaling pathways [35,36,123]. Interestingly, the flowers of *atmyb21* mutant plants showed a delay in recovery of filament elongation under drought treatment compared to wild type plants, suggesting that AtMYB21 can contribute to the recovery in the drought-treated flowers and that it is important for the maintenance of fertility. The authors speculated that AtMYB21 could play a role in the integration of different hormonal pathways, particularly in the possible crosstalk between JA/GA and ABA in drought response [34].

The chrysanthemum (*Chrysanthemum morifolium* Ramat.) *CmMYB2* gene is involved in abiotic stress response and flowering time modulation [37]. Its expression in leaves was up-regulated in response to osmotic, salt and cold treatments, and by the application of exogenous ABA. In *Arabidopsis*, *CmMYB2* ectopic expression increased plant sensitivity to ABA, reduced stomatal aperture, improved tolerance to drought and salt stress, and induced the expression of several stress responsive-genes, such as *AtRD22*, *AtRD29A*, *AtRAB18*, *AtCOR47*, *AtABA1* and *AtABA2*. Transgenic *Arabidopsis* plants expressing *CmMYB2* were delayed in flowering. Moreover, the expression of the genes *CONSTANS* (*AtCO*), *FLOWERING LOCUS T* (*AtFT*), *SUPPRESSOR OF OVEREXPRESSION OF CONSTANS1* (*AtSOC1*), *LEAFY* (*AtLFY*) and *APETALA1* (*AtAP1*) was down-regulated. As these genes are key regulators of the four pathways (photoperiod, vernalization, autonomous and GA-dependent pathways) that control flowering time in *Arabidopsis* [124], these data showed that *CmMYB2* overexpression results in complex changes in the flowering regulatory network, suggesting a role for *CmMYB2* in this process [37].

## 3. Metabolite Biosynthesis

### 3.1. Cell Wall Components

Plants exposed to water deficit display morphological changes that are the result of plant cell wall modifications. It was reported that cellulose, hemicellulose, pectins and lignin synthesis can be altered in response to water deficit, as recently reviewed [125].

Although different MYB proteins have been described as regulators of lignin, xylan and cellulose biosynthesis, as recently reviewed [126], a role in the response to drought and salt stress was described only for AtMYB52 [38]. An ABA-hypersensitive mutant was isolated during a screening of an activation tagging mutants collection. The mutation was mapped in the *AtMYB52* locus. The activation tagged mutant and lines overexpressing the gene under the control of the *CaMV35S* promoter displayed ABA-hypersensitivity, increased drought tolerance and salt sensitivity compared to wild type plants. These results suggest that *AtMYB52* overexpression does not affect osmotic response. Transcriptional data showed that *AtMYB52* overexpression affected the expression of genes involved in secondary cell wall biosynthesis, confirming previous data [126], and also genes involved in ABA biosynthesis and response. Hence, two possible models were suggested to describe the AtMYB52 role: changes in cell wall architecture, caused by *AtMYB52* overexpression, may trigger ABA-hypersensitivity; alternatively, AtMYB52 may directly affect ABA metabolism and response [38].

### 3.2. Cuticle and Suberin

Plants have specialized lipid-based barriers that protect them from various stresses, such as drought and pathogen attack: cuticle, that covers the outer wall of epidermal cells on aerial organs, and suberin, mostly present on the inner face of primary walls of certain boundary tissue layers of plants, such as root endodermis, root and tuber peridermis, and seed coats [127].

The cuticle is synthesized by the epidermis of fruits, leaves, primary stems and organs of flowers. It is primarily comprised of cutin, a glycerolipid polymer and associated waxes. Among its different defense roles, it protects leaves and stems from non-stomatal water loss [127]. Cuticular wax accumulation is closely associated with drought tolerance response, as well supported by different studies ([40] and references therein).

To date, different *Arabidopsis* R2R3-MYB TFs that regulate cuticle components biosynthesis have been reported [128], but a role also in drought response was suggested only for AtMYB94 and AtMYB96 [39,40].

AtMYB96, already described for its role in regulation of lateral root growth and stomatal movements (Section 2.1 and Section 2.3), and AtMYB94, closely located in the phylogenetic tree [39], have been characterized as transcriptional regulators of the cuticular wax biosynthetic genes [39,40]. These two genes share different features: (i) their expression is higher in stem epidermal peels than in stem [39]; (ii) they are up-regulated in response to ABA, drought, and salinity stress [15,39]; (iii) they are able to up-regulate the transcription of cuticular wax biosynthetic genes, although they activate distinct target genes, with the exception of *KCS2/DAISY* gene (*KCS1*, *KCS2*, *KCS6*, *KCR1*, and *CER3* are direct targets of AtMYB96, while *WSD1*, *KCS2/DAISY*, *CER2*, *FAR3*, and *ECR* genes are targets of AtMYB94); (iv) in *AtMYB94*- and *AtMYB96*-overexpressing plants the total wax loads increase in leaves as compared to those of wild type plants, and at a lower level also in stems, even if this effect is not so evident, probably because total wax load in this structure is already high [39]; and (v) the rate of cuticular transpiration in leaves of plants overexpressing one of the two genes was reduced under drought stress [15,39]. Moreover, a knockout mutant for *AtMYB96* was characterized and it showed the opposite phenotype to the overexpressing lines, particularly the down-regulation of the wax biosynthetic genes and the decrease by 34% of total wax load [15]. For AtMYB96 a role in pathogen resistance was also described [74] (Section 4.2), probably dependent on its activity as a regulator of cuticular wax accumulation [39]. Hence, AtMYB96 and AtMYB94 TFs may act as master transcriptional activators of wax biosynthesis and accumulation in response to drought. Moreover, this regulatory mechanism seems to be conserved among species [41,42]. The ectopic expression of *AtMYB96* in *Camelina sativa*, an emerging oilseed crop, gave very similar results to the overexpression of *AtMYB94* or *AtMYB96* in *Arabidopsis* [41]. In fact, Camelina plants expressing *AtMYB96* exhibited a strong up-regulation of wax biosynthesis and increased accumulation of wax load by approximately 50% relative to the wild type ones. Consequently, they showed decreased cuticular transpiration and increased drought tolerance. In addition, transgenic plants exhibited an increase in the levels of alkanes and primary alcohols by approximately 2-fold and 1.5-fold, respectively, suggesting a role for this TF also in the synthesis regulation of these compounds. This function may be exploited for the production of lubricants, adhesives, coatings, sealants, impregnation materials, candles, and cosmetics [41].

The EsWAX1 protein from *Eutrema salsugineum* shared 86% amino acid similarity with AtMYB96 [42]. *E. salsugineum* (formerly *Thellungiella halophila*), known as saltwater cress, is widely used as a halophytic model for stress-tolerance research in plants and it is closely related to the model species *A. thaliana.* The recent comparison of its genome to that of *Arabidopsis* revealed that the stress-tolerance of *E. salsugineum* is unlikely to be determined by variation in few genes, but rather to a global network adjustment of multiple regulatory mechanisms (transcriptional, post-transcriptional, translational, and post-translational systems) may be responsible for its adaptation to extreme environments [129]. The *EsWAX1* transcript was significantly activated in response to drought and ABA, suggesting that it may play a crucial role in ABA-mediated drought stress response. The ectopic expression of *EsWAX1* in *Arabidopsis*, under the control of the constitutive *CaMV35S* promoter, increased the expression of several wax-related genes such as *CER1*, *KCS1* and *KCR1* and consequently the accumulation of cuticular wax. In addition, the expression of ascorbic acid biosynthetic genes (*VTC1*, *GKLDH* and *MIOX4*) was up-regulated in the *EsWAX1*-expressing lines and was accompanied by an increase in ascorbic acid content, suggesting a role for EsWAX1 also in the regulation of biosynthesis of ascorbic acid, whose antioxidant activity is associated with increased tolerance to abiotic stress [130]. However, *EsWAX1* ectopic expression caused negative pleiotropic effects, as 65% of transgenic lines were dwarf with leaves smaller than those of wild type plants. In order to overcome these adverse effects on plant growth, this gene has been expressed under the control of the drought stress-inducible *RD29A* promoter, a strategy already successfully used for the induced expression of other TFs regulating stress response (see for example [131]). Under standard growth conditions the *RD29A::EsWAX1* plants showed no differences compared to wild type plants. Interestingly, these plants showed an improved drought tolerance in term of increased survival rate after recovery from a dehydration treatment and reduced water loss of detached leaves [42].

Suberin is a glycerol-based polymer consisting of a polyaliphatic polyester linked with phenolic components and embedded waxes [127]. As previously mentioned, unlike cuticle, suberin is mainly deposited on the inner cell wall of specific cell types such as endodermal and peridermal cells.

The *AtMYB41* gene has been recently described as a regulator of suberin synthesis and assembly [43]. *AtMYB41* expression was strongly up-regulated in response to desiccation, osmotic and high salinity treatments in an ABA-dependent manner [44,45]. Transgenic lines overexpressing *AtMYB41* appeared dwarf, with smaller cells, characterized by an abnormal morphology; these plants showed enhanced sensitivity to desiccation and enhanced permeability of leaf surface. Based on these phenotypic alterations and on the different expression of genes involved in cell expansion and in wax biosynthesis, a possible role of this TF in the regulation of cell expansion and cuticle biosynthesis and deposition was suggested [44]. AtMYB41 is also implicated in conferring salt tolerance during germination in a phosphorylation-dependent manner (via mitogen-activated protein (MAP) kinase activity [46]). However, only very recently, chemical and ultrastructural analysis on *Arabidopsis* plants overexpressing *AtMYB41* revealed both the ectopic production of aliphatic suberin-type polyesters and the deposition of suberin-associated wax-type compounds in the cell wall of leaf epidermal cells, although these components are typical of suberized endodermal and peridermal root cells [43]. Moreover, overexpression of *AtMYB41* in *Arabidopsis* led to the production of atypical leaf wax components such as hydroxycinnamates and monoacylglycerols. The chemical and ultrastructural phenotypes observed in *AtMYB41*-overexpressing lines were also observed when *AtMYB41* was transiently expressed in *N. benthamiana* leaves under the control of the *CaMV35S* promoter. In both systems, an increased synthesis of lignin was described. These results were also confirmed at transcriptional level in *Arabidopsis* transgenic lines, where increased transcription of suberin, lignin and also phenylpropanoid biosynthetic genes was observed [43]. The same authors reported that the *AtMYB41* promoter, fused upstream of the *GUS* reporter gene, drove GUS activity in endodermal and surrounding cortical cells under ABA and salt treatments, but not under standard growth conditions. Although the exact biological function of this TF is not yet clear, strong evidence suggests a role of *AtMYB41* in augmenting aliphatic suberization under conditions of abiotic stress [43].

### 3.3. Flavonoids

Flavonoids represent a major component of secondary metabolism in plants. Their most obvious function is the ability to impart color in flowers and fruits and so attract pollinators and seed dispersers. Flavonoids were extensively described as defense metabolites, which are synthesized in response to both abiotic and biotic stresses, and are supposed to act as antioxidants *in vivo* based on their *in vitro* antioxidant activity [132]. However, only recently, the antioxidant function of flavonoids *in planta* in response to drought and also to oxidative stress was experimentally identified [47]. For this purpose, a series of transgenic and mutant *Arabidopsis* lines, including single and double overexpressors for *AtMYB12*/*PFG1* (*PRODUCTION OF FLAVONOL GLYCOSIDES1*), a flavonoid regulator controlling expression of the early biosynthetic genes, and for *AtMYB75/PAP1* (*PRODUCTION OF ANTHOCYANIN PIGMENT1*), an anthocyanin regulator controlling the last steps of the biosynthesis, were analyzed using transcriptomics, hormonomics and metabolomics. The results showed that the tolerance to oxidative and drought stresses is enhanced in plants that are able to overaccumulate flavonoids characterized by a strong antioxidant activity. Interestingly, the enhanced stress tolerance was solely due to the antioxidant chemical character of overaccumulated flavonoids. It is now demonstrated that flavonoid accumulation is a late response implemented to protect plants in response to abiotic stress exposure [47].

Another MYB protein that regulates anthocyanin biosynthesis, suggested to have a role also in osmotic stress response, is the apple MdMYB10 [48]. Its ectopic expression in *Arabidopsis* caused an improved tolerance to sorbitol-mediated osmotic stress and transgenic plants exhibited a higher accumulation of flavonoids, chlorophyll and proline compared to wild type plants, as well as a lower content of malondialdehyde (MDA), a decomposition product of the oxidation of polyunsaturated fatty acids [49].

The *MYB* genes, which are known to be involved in flavonoid accumulation in fruit, have been usually analyzed for their involvement in fruit ripening without any evaluation under stress conditions [133]. However, a strong correlation between the expression of some *MYB* genes involved in anthocyanin biosynthesis and the flavonoid accumulation in ripening fruit under water stress in the field was reported for grape [134]. These data show that drought actively promotes fruit maturation and anthocyanin biosynthesis, opening new perspectives on the study of MYB functions in the fruit in response to drought.

## 4. Crosstalk among Different Stress Responses

Plant response to environmental cues is dynamic and involves complex crosstalk among different regulatory levels, including adjustment of metabolism and gene expression for physiological and morphological adaptation [135,136].

Various abiotic stresses result in both general and specific effects on plant growth and development. For example, osmotic stress and the associated oxidative stress appear to be common consequences of exposure to drought, salinity and low temperatures. For this reason, plants have evolved multiple stress perception and signal transduction pathways, which may crosstalk at various steps in the pathways, as well as common stress-induced protection/repair strategies, such as the production of dehydrins, chaperonins, osmoprotectants and ROS detoxifying compounds [137].

Moreover, extensive overlap between the signaling pathways governing biotic and abiotic stress responses exists. Plant hormones are key components of defense and adaptation mechanisms and signal interactions can be both synergistic and antagonistic, resulting in positive and negative functional outcomes [138,139].

TFs are of key importance in the signaling cascades and in generating specificity in stress responses. Among them, *MYB* genes are becoming increasingly associated with the control of both biotic and abiotic stress responses, and many of them are reported to play a pivotal role in the crosstalk among abiotic stresses and between abiotic and biotic stresses [8]. Here we describe the *MYB* genes with a well-characterized role in response to drought as well as to other abiotic and/or biotic stresses (Table 2).

**Table 2:** *MYB* genes involved in crosstalk between drought and other stress responses. Flg 22: flagellin 22; HrpN: harpin protein; PEG: polyethylene glycol; Pi: Phosphate; TNV: tobacco necrosis virus.

Gene Name	Abiotic Stresses		Biotic Stresses			Other Stresses
	Salt	Cold/Freezing	Bacteria	Fungi	Insects	
*AtMYB2*	–	–	–	–	–	low oxygen, Pi deficiency
*AtMYB20*	×	–	–	–	–	–
*AtMYB12*	–	–	–	–	*S. litura*	UV-B
*PFG1*					*H. armigera*	flg 22
*AtMYB75*	–	–	–	–	*S. litura*	–
*PAP1*						
* AtMYB44*	×	–	*P. syringae*	–	*M. persicae *	wounding
					*P. xylostella*	
*AtMYB96*	–	×	*P. syringae*	–	–	flg 22
*AtBOS1*	×	×	–	*B. cinerea*	–	oxidative
*MYB108*						
*AtMYB15*	×	×	–	–	*M. persicae*	wounding
						HrpN_Ea_
*OsMYB3R-2*	×	×	–	–	–	–
*OsMYB2*	×	×	–	–	–	–
*OsMYB48-1*	×	–	–	–	–	–
*OsMYB4*	×	×	*P. syringae*	*B. cinerea*	–	oxidative
						TNV
*TaMYB2A*	×	×	–	–	–	–
*TaMYB19*	×	×	–	–	–	–
*TaMYB33*	×	–	–	–	–	–
*TaPIMP1*	×	–	*R. solanacearum*	*B. sorokiniana*	–	oxidative
*GmMYBJ1*	–	×	–	–	–	–
*CpMYB10*	×	–	–	–	–	–
*MdSIMYB1*	×	×	–	–	–	–
*MdoMYB121*	×	×	–	–	–	–
*SpMYB*	×	–	–	*A. alternata*	–	–

### 4.1. Crosstalk among Different Abiotic Stress Responses

More than 20 years ago AtMYB2 was the first MYB TF characterized for its transcriptional induction in response to desiccation, high salt and ABA treatments [140]. A more detailed analysis revealed that this protein and AtMYC2, a bHLH TF, are able to bind to the MYB and MYC recognition sites, respectively, which are present in a specific region of the promoter of *AtRD22* and they are able to transactivate a *GUS* reporter gene fused to this promoter sequence [50]. Plants overexpressing both *AtMYB2* and *AtMYC2* showed a significant reduction in electrolyte leakage in response to mannitol treatment, compared to wild type plants, indicating an improved response to osmotic stress. Moreover, plants overexpressing only *AtMYB2* or both *AtMYB2* and *AtMYC2* were hypersensitive to ABA during germination [51]. Transcriptomic analysis revealed that different osmotic stress-inducible genes were up-regulated in plants overexpressing both TFs under standard growth conditions. AtMYB2 and also AtMYC2 clearly function as transcriptional activators in ABA-inducible gene expression under drought stress in plants. However, plants overexpressing *AtMYB2*, grown on soil, exhibited severe growth inhibition, dependent on smaller cell size and the phenotype was even smaller when *AtMYC2* was also overexpressed, while plants grown on GM agar plates were similar to the wild type ones [51]. AtMYB2 was also characterized for its role in the response to other stresses. It was suggested that it is a key regulatory factor in the induction of the *ADH1* (*ALCOHOL DEYHDROGENASE1*) gene by low oxygen in the roots [52]. More recently, it was shown that AtMYB2 directly binds to a MYB-binding site located in the promoter of *miR399f* and activates the expression of this *Arabidopsis* miRNA gene [53]. The *miR399* expression is strongly induced by phosphate (Pi) starvation in vascular tissues of the shoot, then mature *miR399* is translocated to the root where it leads to the degradation of PHOSPHATE2, an important central negative regulator of the Pi response pathway, triggering the restoration of Pi homeostasis [141]. Both *AtMYB2* and *miR399f* are expressed mainly in vascular tissues of cotyledons and in roots. Compared to wild type plants, *AtMYB2* overexpressing lines showed a higher reduction of primary root growth under Pi-deficiency conditions and a major increase in root hair density under Pi accumulation. Moreover, these plants showed increased expression of some Pi starvation-responsive genes. These results indicate that AtMYB2 is part of the Pi signaling pathway for maintaining Pi homeostasis within plants [53]. AtMYB2 has also a role in the regulation of the post apical dominance mechanism by which *Arabidopsis* inhibits the outgrowth of axillary buds. The *atmyb2* T-DNA insertion lines displayed enhanced expression of cytokinin-synthesizing isopentenyltransferases genes, higher levels of cytokinins compared to wild type, and a bushy phenotype at late stages of development. As a result of the continuous generation of new shoots, *atmyb2* plants have a prolonged life span. These data showed that AtMYB2 is able to regulate whole plant senescence through the inhibition of cytokinin-mediated branching at late stages of development [142]. All the information collected on AtMYB2 function suggest that this TF may be a linker among the abiotic stress responses, Pi homeostasis and developmental regulation [143].

The *AtMYB20* gene, previously mentioned for its role in the regulation of stomatal opening (Section 2.3), is involved in the response to salt and drought stresses [26,54]. Cui and co-workers [54] showed that its expression was induced by NaCl (250 mM for 2 and 4 h) and ABA (200 µM for 4 h) treatments as well as by long-term (six days) drought stress, whereas it was repressed by the presence of SA and MeJA [54]. In a subsequent study, an expression analysis confirmed the *AtMYB20* induction following NaCl treatment (300 mM for 1.5 and 3 h), but showed some differences in drought and ABA-mediated induction: desiccation (for 1 and 2 h), cold (4 °C for 6 and 12 h), and ABA (100 μM for 1.5 and 3 h) treatments repressed the expression level of *AtMYB20* [26]. Cui and co-workers [54] generated transgenic *Arabidopsis* lines overexpressing or repressing *AtMYB20*. The plants overexpressing *AtMYB20* exhibited an enhanced salt stress tolerance while repression lines were more vulnerable to NaCl than wild type plants. Under control conditions, all the plants overexpressing or repressing *AtMYB20* showed a slight reduction of the expression of *AtABI1*, *AtABI2* and *AtPP2CA* genes compared to wild type plants. These genes encode 2C serine/threonine protein phosphatases (PP2Cs), which negatively regulate ABA signaling. After NaCl treatment, the salt-induced expression of these genes in wild type plants was significantly suppressed in overexpressing lines and enhanced in repression lines. These data, along with the evidence provided by the authors that AtMYB20 is able to bind the MYB recognition sequence in the promoter regions of *AtABI1* and *AtPP2CA*, suggested that AtMYB20 negatively regulates the expression of PP2Cs, the negative regulators of ABA signaling, and enhances salt tolerance, through a possible interaction with other TFs [54]. Conversely, *AtMYB20*-repression lines generated by Gao and co-workers [26] were more tolerant to desiccation and hypersensitive to ABA than wild type, whereas *AtMYB20* overexpression lines displayed a similar behavior to wild type plants. Moreover, *atmyb20* knockout mutation intensified stomatal closure in the presence of ABA, while *AtMYB20*-overexpressing plants displayed insensitivity to ABA-dependent stomatal closure. Furthermore, expression of ABA-responsive genes such as *AtABI3*, *AtABI4*, *AtABI5*, *AtABF3*, and *AtABF4* was more evident in *atmyb20* plants than in wild type, whereas expression of these genes in *AtMYB20*-overexpressing plants was depressed. These data suggested that *AtMYB20* negatively interacts with genes involved in ABA synthesis or signaling under desiccation stress, in accordance with expression data of Cui and co-workers [54] on their *AtMYB20*-overexpressing lines, where a reduction of the expression of ABA-responsive genes (e.g., *AtABI1* and *AtABI2*) was observed in control conditions. Furthermore, an expression analysis conducted by Gao and co-workers [26] on *AtABI1*, *AtABI2* and *AtAtPP2CA* genes indicated that their transcript level was significantly reduced in overexpressors and increased in mutant lines. Although the data provided by these two papers are not completely in accordance, an involvement of *AtMYB20* in the ABA signaling pathway and in the response to salt and desiccation stresses is clear. Further analyses can better elucidate its role in these responses.

In rice, one of the first identified genes involved in the crosstalk among different abiotic stresses is *OsMYB3R-2*, which contains three imperfect repeats in the DNA-binding domain [9]. Its expression was induced by cold, drought, and salt stresses. *Arabidopsis* transgenic plants expressing *OsMYB3R-2* showed a little retarded growth under normal conditions and an increased tolerance to freezing, drought, and salt stresses. Moreover, germination of *OsMYB3R-2*-expressing seeds was insensitive to ABA and NaCl treatments. The elevated stress tolerance of *OsMYB3R-2* expressing plants coincided with the up-regulation of stress-responsive genes, including *AtDREB2A*, *AtCOR15a*, and *AtRCI2A*. *AtCOR15a* and *AtDREB2A* are involved in stress signaling by the CBF/DREB1 pathway, and *AtRCI2A* by the CBF/DREB1-independent pathway. These data indicated that *OsMYB3R-2* acts as a master switch in stress tolerance by activating different pathways [9]. A following study showed that OsMYB3R-2 acts in rice not only in stress response, but also in processes related to cell cycle regulation [55]. OsMYB3R-2 specifically bound to the MSA-element, present in the promoter of the cyclin *OsCycB1;1* gene, and the overexpression of *OsMYB3R-2* in rice induced a higher expression level of several G2/M phase-specific genes and an increased cell mitotic index compared to *OsMYB3R-2*-antisense lines or wild type plants in response to cold treatment. Further data on transgenic lines overexpressing both *OsMYB3R-2* and *OsCycB1;1* indicated that OsMYB3R-2 targets *OsCycB1;1* and regulates the cell cycle progression during chilling stress, suggesting the existence of a cold tolerance mechanism in rice which acts through the regulation of the cell cycle and which is controlled by key genes including *OsMYB3R-2* [55].

Another rice *MYB* gene, *OsMYB2*, which is involved in cold, drought, and salt stresses has been recently identified [56]. OsMYB2 is localized in the nucleus and its transactivation activity has been demonstrated. The expression of *OsMYB2* was induced by salt, cold, PEG-mediated osmotic treatments and by ABA exogenous application. Analyses of transgenic rice plants *OsMYB2*-overexpressing or repressing *OsMYB2* through the RNAi technique, showed that *OsMYB2* is able to enhance tolerance to low temperatures, NaCl and PEG-mediated osmotic/drought treatments in hydroponic culture or under soil growth conditions. Moreover, the analyses showed that *OsMYB2*-overexpressing lines were more sensitive to ABA than wild type and RNAi lines. An in-depth analysis was conducted to dissect the role of *OsMYB2* in the response to salt stress. Upon exposure to salt stress, *OsMYB2*-overexpressing plants showed a higher increase in the content of proline and soluble sugars, in the expression level of genes involved in proline biosynthesis and transport, and in the activities of POD, SOD, and CAT compared to wild type plants, as well as a lower increase in H_2_O_2_ and MDA content. These data suggested that the overexpression of *OsMYB2* confers greater tolerance to the oxidative stress associated with salt stress through the activation of an efficient antioxidant system. Moreover, the ectopic expression of *OsMYB2* induced the transcript level of the TF-encoding gene *OsDREB2A* and of the genes *OsLEA3* and *OsRAB16A*, which code for two late embryogenesis abundant proteins implicated in abiotic stress response, suggesting that OsMYB2 may regulate the expression of *LEA* genes through the OsDREB2A-dependent signaling pathway. Finally, microarray data indicated that *OsMYB2*-overexpression also led to changes in the transcript level of numerous genes involved in stress response, confirming that OsMYB2 plays an important role in stress tolerance in rice by regulating a number of downstream genes that are closely associated with plant tolerance to abiotic stress [56].

The rice *OsMYB48-1* gene plays a positive role in drought and high salinity tolerance by regulating stress-induced ABA synthesis genes [57]. Its overexpression in rice significantly improved tolerance to osmotic (mannitol, PEG), salt and drought stresses. Overexpression plants were hypersensitive to ABA and, under stress conditions, they showed a reduced rate of water loss, lower MDA content, higher proline content and higher ABA accumulation than wild type plants. Moreover, the overexpression of *OsMYB48-1* induced the expression of some ABA biosynthesis (*OsNCED4*, *OsNCED5*), early signaling (*OsPP2C68*, *OsRK1*) and late responsive (*OsRAB21*, *OsLEA3*, *OsRAB16C* and *OsRAB16D*) genes under drought stress compared to wild type plants. These data indicated that *OsMYB48-1* regulates the expression of ABA synthesis genes thus leading to increased endogenous ABA accumulation under drought stress conditions [57]. Moreover, a previous work demonstrated that TSFR1, a tomato ethylene response factor (ERF) protein, enhanced osmotic and drought tolerance of rice by modulating the increase in stress responsive gene expression, including several *MYB*, *MYC* and proline synthesis and photosynthesis-related genes [58]. Interestingly, one of these *MYB* genes was *OsMYB59*, which is reported with the same ID as *OsMYB48-1* (LOC_Os01g74410/Os01g0975300), confirming the important role of this gene in the osmotic stress response.

In wheat, several *MYB* genes involved in the multiple responses to abiotic stresses have been identified. Three *TaMYB2* members were identified and designated *TaMYB2A*, *TaMYB2B*, and *TaMYB2D* based on their genomic origins [59]. The *cis*-regulatory elements in the promoter regions were compared, and their expression patterns under different abiotic stress conditions were analyzed. In particular, *TaMYB2A* was early induced by NaCl, cold and PEG-mediated osmotic treatment, whereas it was induced by exogenous ABA only 12 h after its application. Subcellular localization analysis indicated that TaMYB2A is located in the cell nucleus. Transgenic *Arabidopsis* plants expressing *TaMYB2A* displayed no morphological differences compared to wild type plants, with the exception of an anticipation in flowering time of about 3–5 days. Transgenic plants showed a reduced water loss rate for detached rosettes and a lower stomatal conductance than wild type plants. *TaMYB2A*-expressing *Arabidopsis* plants exhibited higher cell membrane stability and a higher photosynthetic potential under salt stress, compared to wild type plants, as well as a pronounced tolerance to drought, salt and freezing stresses. An expression analysis indicated that the transcript level of six genes involved in abiotic stress response (*AtDREB2A*, *AtRD29B*, *AtRD22*, *AtCOR15*, *AtRab18* and *AtABI2*) were consistently higher in stressed or non-stressed transgenic plants compared to wild type plants, confirming a role of *TaMYB2A* in the response to multiple abiotic stresses [59]. *TaMYB2A* is the putative ortholog of *OsMYB4* in wheat [82]. As *OsMYB4* has a master role in abiotic and biotic stress responses (see Section 4.2), it could be of interest to further analyze the response to biotic stresses of TaMYB2A.

Similar analyses have been recently conducted for the *TaMYB19* gene: the isolation and characterization of the three homologous copies *TaMYB19-A*, *TaMYB19-B* and *TaMYB19-D* were reported. Moreover, a functional characterization of the *TaMYB19-B* gene revealed its potential role in the tolerance to abiotic stresses [60]. Transgenic *Arabidopsis* lines expressing the *TaMYB19-B* gene exhibited an increased tolerance to salt, drought and freezing stresses compared to wild type plants. Changes in physiological indices were evaluated, and a higher soluble sugar content, a lower electrolyte leakage and a lower MDA content were found in transgenic seedlings compared to wild type seedlings under all the abiotic stress treatments. However, significant variations in proline content between transgenic and wild type seedlings were observed only under mannitol-mediated osmotic and freezing treatments. An expression analysis revealed that the genes *AtRD29A*, *AtRD22* and *AtMYB2* exhibited a significantly higher expression level in the *TaMYB19-B* transgenic plants compared to wild type, confirming the involvement of this gene in the tolerance response to abiotic stresses [60].

*TaMYB30-B* is the homologous sequence in the chromosome 2B of *TaMYB30* and is involved in response to osmotic stress. Its expression was induced by PEG treatment [61]. *Arabidopsis* transgenic plants expressing *TaMYB30-B* exhibited a greater level of seed germination under mannitol-mediated osmotic stress, and an enhanced tolerance to drought stress at the seedling stage. Under osmotic stress conditions, transgenic plants showed a higher accumulation of proline and soluble sugars, a lower amount of MDA and a lower water loss rate compared to wild type plants. Moreover, the stress-responsive genes *AtRD29A* and *AtERD1* exhibited a significantly higher expression level in transgenic plants under normal conditions [61]. Although *TaMYB30-B* has been examined only for its role in drought response, the changes in the physiological indices here described suggest an involvement of this gene in the response to other abiotic stresses. Further analyses can support this hypothesis.

The *TaMYB33* gene was induced by NaCl, PEG and ABA treatments and its overexpression in *Arabidopsis* increased tolerance to NaCl treatment, drought and mannitol-mediated osmotic stress [62]. Expression analysis of stress-responsive genes involved in ABA-signaling, osmotic balancing and ROS scavenging indicated that in *Arabidopsis TaMYB33*-expressing plants, ABA synthesis was elevated while its signaling was restricted, and the improved salt and drought tolerance could be due to a superior ability to balance the osmotic pressure and to detoxify ROS produced under stress [62].

Recently, a *MYB* gene involved in the response to cold and drought stresses was isolated from soybean (*Glycine max* (L.) Merr.) and designated as *GmMYBJ1* [63]. An expression analysis under abiotic stress conditions was conducted, revealing a clear *GmMYBJ1* induction under cold and osmotic stress and also under NaCl and ABA treatments, though with some fluctuations during these latter treatments. Transgenic *Arabidopsis* plants expressing *GmMYBJ1* showed an improved drought and cold tolerance compared to wild type plants, in terms of growth inhibition and survival rate. When subjected to osmotic stress, transgenic plants displayed also a higher seed germination rate and a lower increase of MDA content than wild type plants, as well as a lower water loss rate for detached leaves. When subjected to cold stress, transgenic plants showed a higher content of soluble sugars than that of wild type plants. An expression analysis indicated that the transcript level of some stress-related genes (*AtRD29B*, *AtCOR47*, *AtCOR78*, *AtCOR15a* and *AtP5CS*) was up-regulated in the *GmMYBJ1*-expressing plants compared to wild type plants, whereas the expression level of *AtDREB2A* was slightly down-regulated [63].

The so-called resurrection plants exhibit protoplasmic desiccation tolerance, as they can withstand long periods with air of 0% (*v*/*v*) relative humidity, reviving a few hours after exposure to water. The best-characterized example is *Craterostigma plantagineum* Hochst., a South African plant living on rocks in shallow soil [144]. The gene *CpMYB10*, which has been isolated from this plant, encodes a R2R3-type MYB TF [64]. Its expression was rapidly induced by desiccation and ABA treatment in both leaves and roots. The ectopic expression of *CpMYB10* conferred stress tolerance in *Arabidopsis* transgenic plants that displayed a higher germination rate compared to wild type plants under osmotic stress conditions, as well as an increased tolerance to drought and salt stress. Moreover, overexpression of *CpMYB10* in *Arabidopsis* conferred glucose insensitivity and ABA hypersensitivity during seed germination. An expression analysis on several stress-responsive genes revealed that the transcript level of two genes encoding hydrophilic proteins, *AtRD29a* and *AtCor15a*, and of the alcohol dehydrogenase *AtADH1* gene was higher in *CpMYB10* expressing plants compared to wild type plants upon ABA treatment. Moreover, the transcript level of *AtRD22*, *AtCor15a* and *AtP5CS1* was lower in transgenic plants compared to wild type plants under control conditions, suggesting that CpMYB10 might act on these genes as repressor or activator. Taken together, these data indicate that *CpMYB10* in *Arabidopsis* is able to confer stress tolerance and alter ABA and glucose signaling responses [64].

Few *MYB* genes involved in the response to different stresses have been identified in tree species.

A genome wide analysis of apple *MYB* genes allowed the identification of the *MdoMYB121* gene, which is involved in the tolerance to multiple abiotic stresses [65]. An expression analysis under different treatments (NaCl, ABA, PEG and cold) was conducted on 18 apple *MYB* genes to evaluate their possible involvement in the response to abiotic stresses. Interestingly, the transcript level of six genes (*MdoMYB22*, *121*, *146*, *148*, *155*, *206*) increased in response to all the treatments. The role of *MdoMYB121* was further investigated. Transgenic tomato plants expressing *MdoMYB121* showed a better performance compared to wild type plants when exposed to salt, drought and cold stresses. In particular, under salt stress transgenic plants accumulated a lower Na^+^ content and exhibited a lower Na^+^/K^+^ ratio than wild type plants, indicating that *MdoMYB121* is involved in the regulation of the physiological balance between Na^+^ and K^+^ in response to salt stress. Under drought conditions, transgenic plants exhibited a greater ability to maintain water content than wild type plants, suggesting that the overexpression of *MdoMYB121* enhances drought tolerance at least partially by reducing the water loss. *MdoMYB121* expressing tomato plants exhibited lower levels of electrolyte leakage and MDA and a higher content of the osmoprotectant proline than wild type plants in response to salt, drought, and cold stresses. Furthermore, transgenic apple plants overexpressing *MdoMYB121* exhibited a higher tolerance to salt, drought and cold stresses compared to wild type plants. These data clearly indicated that *MdoMYB121* is able to improve tolerance to these abiotic stresses, through the reduction of the membrane damage and the accumulation of protective compounds [65].

Recently, a *MYB* gene has been isolated from trifoliate orange (*Poncirus trifoliata* (L.) Raf.) and designated as *PtsrMYB* [66]. The transcript level of *PtsrMYB* was up-regulated by dehydration, salt, cold and ABA treatments, with differences depending on the time of the treatments. Tobacco transgenic plants expressing *PtsrMYB* showed enhanced dehydration tolerance and less water loss, as well as lower levels of MDA and ROS compared to wild type plants. Moreover, a higher expression level of two arginine decarboxylase (*ADC*) genes, which play a critical role in putrescine accumulation under abiotic stress, and a greater level of free polyamines, especially putrescine, were observed before and after dehydration treatment [66]. It has been suggested that polyamines have antioxidant effects, through the binding of anions and cations, thus inhibiting lipid peroxidation and metal-catalyzed oxidative reaction and ameliorating ROS-derived oxidative stresses [145]. These data suggested that PtsrMYB positively regulates the expression of the *ADC* genes, thus activating the ADC enzyme, which stimulates putrescine biosynthesis and consequently the synthesis of downstream spermidine and spermine [66].

### 4.2. Crosstalk among Abiotic and Biotic Stress Responses

*Arabidopsis MYB* genes regulating flavonoid biosynthesis, recently shown to have a direct role in drought response, were also implicated in response to biotic stress. Tobacco plants overexpressing *AtMYB12* exhibited increased resistance to *Spodoptera litura* and *Helicoverpa armigera* moths. The enhanced accumulation of rutin, toxic for herbivores, is considered the most probable cause of this tolerance phenotype [67]. Tobacco plants overexpressing *AtMYB75* (*PAP1*) showed greater tolerance to *S. litura*. There was a clear positive correlation between the level of anthocyanin compounds accumulated and the tolerance response to the insect. The PAP1-stimulated anthocyanin accumulation was attenuated by herbivory [68]. Interestingly, it was reported that *AtMYB12* is also involved in a precise mechanism of crosstalk between abiotic and biotic stresses [69]. Particularly, in *Arabidopsis* cell suspension culture, ultraviolet-B (UV-B) light induced the flavonol gene transcription and consequently the flavonol production. The accumulation of these compounds was reduced when the bacterial flagellin peptide elicitor flg22 was concurrently applied. At the same time, flg22 enhanced the production of defence-related compounds (phytoalexin, camalexin, scopoletin and lignin). Flavonols, lignin and scopoletin all derive from phenylalanine. When both stresses (UV-B and flg22) are simultaneously applied, the pathway for defence-related compounds increased at the expense of flavonol synthesis [69]. The transcription of *AtMYB12*, a positive regulator of the flavonol pathway, is induced by UV-B and repressed by flg22 treatment, while the expression of *AtMYB4*, a negative regulator of the same pathway [146], is controlled in the opposite way [69]. The authors proposed a model in which AtMYB12 and AtMYB4 antagonistically regulate flavonol pathway genes in response to UV-B and flg22 [69].

An *Arabidopsis* R2R3-type *MYB* gene well characterized for its role in the response to different abiotic and biotic stresses is *AtMYB44*/*AtMYBR1* [32,70,71,72,73,147,148,149]. As already mentioned in Section 2.3, the overexpression of this TF in *Arabidopsis* enhances stomatal closure with the consequent increased drought and salinity tolerance of these transgenics compared to wild type and *atmyb44* mutant plants [32]. However, in early stages of vegetative growth, rosette leaves of *35S:AtMYB44* plants were smaller and prostrate compared to wild type plants. Concerning the involvement of AtMYB44 in the regulation of salinity response, it was shown that *AtMYB44* is a direct and immediate component in the MKK4–MPK3 signaling pathway, known to initiate the adaptation response to numerous abiotic and biotic stresses [70]. It was suggested that the major role of AtMYB44 consists in the activation of genes involved in the prevention of ROS accumulation [147]. Interestingly, transgenic soybean lines overexpressing the *AtMYB44* gene exhibited significantly enhanced drought/salinity tolerance [71]. Under field conditions, these plants showed reduced growth, but much higher yields upon seed harvest. These soybean seeds did not have alterations in their amino acid and fatty acid composition. These data suggest that AtMYB44 activates a tolerance mechanism that is conserved in *Arabidopsis* and soybean [71]. However, other authors showed data on drought response of *Arabidopsis* transgenic plants overexpressing *AtMYB44* in contrast with the ones described till now. In fact, Jaradat and co-workers reported that *Arabidopsis* plants overexpressing *AtMYB44* were more susceptible to injury under water stress than wild type and conversely mutants were more tolerant to water stress and exhibited reduced rate of water loss from leaves [72]. To mimic drought stress, these authors used PEG, which maintains a specific soil water potential, while in the experiment performed by Jung and collaborators water was halted [32]. Jaradat and collaborators hypothesized that the reduced size of the transgenic plants, due to slower growth of above ground tissues and shorter primary roots, is associated with reduced water use and slower depletion of soil moisture, a phenomenon that produces an apparent increase in drought tolerance. These authors concluded that AtMYB44 is a negative regulator of ABA, drought stress and also wounding responses. AtMYB44 appeared to be involved in feedback maintenance of adult, pre-senescent growth, especially under conditions of drought stress and wounding [72]. Similar microarray results were reported in both papers [32,72], showing that many well-known positive effectors or regulators of stress responses were similarly down-regulated in AtMYB44-overexpressors compared to wild type plants [32,72]. Recently, it was shown that AtMYB44 competes with ABI1 phosphatase, a negative regulator of ABA signaling, for the binding to the RCAR1/PYL9 ABA receptor [73]. The proposed model suggested that, upon ABA perception, AtMYB44 competes with ABI1 for binding to RCAR1/PYL9, releasing the inhibitory effect of RCAR1/PYL9 on ABI1 activity and thus down-regulating the expression of ABA-responsive genes [73]. This work reasonably supports the physiological observations from Jaradat and co-workers [72]. AtMYB44 is part of the response networks to ABA/abiotic stress and to wounding/abscission, both of which involve senescence responses. AtMYB44 acts as a negative regulator (feedback repressor) of responses to stress, wounding and abscission in favor of normal growth and development. AtMYB44 has also been extensively characterized for its role in biotic stress response. In particular, it is an integrator of crosstalk between SA and MeJA in plant defense mechanisms. *Arabidopsis* plants overexpressing *AtMYB44* showed up-regulation of genes for SA-mediated defense and enhanced response to the biotrophic pathogen *Pseudomonas syringae* pv. tomato DC3000. On the other hand, in these plants the MeJA-mediated defense response against the necrotrophic pathogen *Alternaria brassicicola* was down-regulated. The knockout mutant *atmyb44* showed opposite effects [148]. These data indicate that AtMYB44 is a positive regulator of SA-response but a negative regulator of MeJA response. AtMYB44 also plays a critical role in *Arabidopsis* resistance to insects such as the phloem-feeding generalist green peach aphid (*Myzus persicae*) and leaf-chewing specialist caterpillar diamondback moth (*Plutella xylostella*) [149]. All these data support the idea that AtMYB44 is a key integrator of different signals and has a major role in the crosstalk between them.

AtMYB96, previously described for its role in drought response through the regulation of lateral root growth, stomatal opening and cuticle components accumulation (Section 2.1, Section 2.3 and Section 3.2), was also characterized for its positive regulation of freezing [75] and pathogen responses [74]. Its overexpression in *Arabidopsis* enhanced freezing and drought tolerance through the transactivation of *LIPID-TRANFER PROTEIN3* (*LTP3*) gene, a direct target of AtMYB96 protein, as shown *in vitro* and *in vivo* assays [75]. Furthermore, AtMYB96 plays a key role to link the ABA-mediated abiotic stress signal with SA biosynthesis and pathogen resistance response [74]. The *myb96-1d* activation tagging line showed enhanced disease resistance to a virulent *Pseudomonas syringae* DC3000 strain, while the *myb96-1* mutant had the opposite phenotype. *AtMYB96* expression was up-regulated very early (within one hour) in response to the treatment with flg22, which efficiently triggers plant defense response, before the induction of the *Pathogen Related* (*PR*)*-1* and the SA biosynthetic *SALICYLIC ACID INDUCTION DEFICIENT2* (*SID2*) genes. *PR-1*, *PR-2*, *PR-5* and *SID2* were highly up-regulated in *myb96-1d* plants. Consistent with these results, the endogenous concentration of SA and SA-β-glucoside were elevated in the same plants. *SID2* was also induced by ABA, drought, osmotic stress and high salinity, even if the inductive effects were reduced in the *myb96-1* mutant, indicating that AtMYB96 is required for *SID2* activation in response to abiotic stress. These data clearly indicate that AtMYB96-mediated ABA signals enhance plant disease resistance by inducing SA biosynthesis [74].

The *Arabidopsis AtMYB108* gene has been mentioned above (Section 2.4) for its role in the regulation of filament elongation and anther dehiscence via the JA and GA signaling pathways [35,123]. *AtMYB108*, closely related to *AtMYB2*, is also one of the first identified *MYB* genes involved in the crosstalk among abiotic and biotic stresses [76]. It was isolated based on a T-DNA insertion allele that resulted in increased susceptibility to infection by the fungal pathogens *Botrytis cinerea* and *Alternaria brassicicola*. For this reason, this gene was designated *BOS1* (*BOTRYTIS_SUSCEPTIBLE_1*). Its expression was induced by *Botrytis* infection, whereas it was blocked in the JA-insensitive *coi1* mutant, suggesting that *AtMYB108/BOS1* may play a role in the defense response regulated by JA. Moreover, the expression of the *PR-1* gene, which is a molecular marker for the systemic acquired resistance (SAR) response, was induced more strongly in the *bos1* background than in the wild type plants. The greater *PR-1* expression can be attributed to the more abundant proliferation of the pathogen in the mutants compared to the wild type plants. These data clearly demonstrate a role for *AtMYB108/BOS1* in resistance to *Botrytis*. Interestingly, *bos1* mutant was sensitive to abiotic stresses, such as high salinity, drought, cold, osmotic, and oxidative stresses. Since ROS have been implicated in signaling in response to both pathogens and abiotic stresses, these data suggested that *AtMYB108/BOS1* could regulate the responses to ROS-mediated signaling from both abiotic and biotic stresses [76].

Another well-characterized gene that is involved in the response to different stresses is *AtMYB15*. Its expression is strongly induced under desiccation [31,82]. *AtMYB15* overexpression lines displayed improved drought and salt stress tolerance, hypersensitivity to exogenous ABA, as well as a higher transcript level of ABA-responsive genes and genes involved in ABA biosynthesis and signaling, suggesting a role of this gene in the ABA signaling pathway [31]. *AtMYB15* is also involved in the response to low temperature: its expression was slightly induced under cold (4 °C) stress and strongly induced by freezing temperatures (0 °C) [77,78]. AtMYB15 was able to interact with ICE1 and bound to MYB recognition sequences in the promoters of CBF genes [78]. The *atmyb15* mutant plants showed increased tolerance to freezing stress, as well as an increased expression of *CBF* genes under cold conditions, whereas its overexpression reduced freezing tolerance and down-regulated the expression of *CBF* genes, suggesting that AtMYB15 negatively regulates *CBF* genes and it is part of a complex network in cold stress response [78]. The cold-mediated induction of *AtMYB15* and *CBF3* seems to be related to the heptahelical protein 1 (HHP1), which is a negative regulator in ABA and osmotic signaling in *Arabidopsis* and seems to be important in the crosstalk between cold and osmotic signaling [79]. Furthermore, a possible role in the response to attack by insects has been suggested [80,81]. The expression of *AtMYB15* was highly induced by wounding and its overexpression in transgenic plants resulted in elevated expression of almost all the genes involved in the shikimate pathway, suggesting that AtMYB15 could be a direct regulator of this pathway in response to wounding [80,82]. Interestingly, *AtMYB15* expression was up-regulated by treatments with ethylene and with the harpin protein HrpN_Ea_, an elicitor secreted by *Erwinia amylovora*. A mutant line generated by T-DNA insertion into the exon region of *AtMYB15* showed a greater susceptibility to the green peach aphid, indicating that AtMYB15 can be important to activate the ethylene defensive pathway [81].

In rice, a master gene playing a pivotal role in the crosstalk among abiotic and biotic stresses is *OsMYB4*. Initially, it was identified as a cold responsive gene [83]. Its ability to positively regulate the transcription of genes involved in the cold response was demonstrated and *Arabidopsi OsMYB4* expressing plants showed a significant increase in cold and freezing tolerance, as well as a change in the expression level of some genes involved in cold response, such as *AtCor15a*, *AtCor78* and *AtPAL2* [83]. Moreover, the ability of *OsMYB4* to induce drought tolerance in *Arabidopsis* transgenic plants was also reported [84]. The *OsMYB4*-expressing plants showed a higher amount of different osmolytes compared to wild type plants, both under normal and stress (cold or drought) conditions, as well as a higher and a lower expression level of the *P5CS* and *P5CD* genes involved in proline biosynthesis and degradation, respectively [84]. A comparative microarray analysis of wild type and *OsMYB4*-expressing *Arabidopsis* plants revealed that *OsMYB4* affects the expression of genes involved in both abiotic and biotic stress responses [85]. Moreover, in *Arabidopsis OsMYB4* improved tolerance/resistance to different abiotic and biotic stress conditions, namely drought, salt, UV, ozone, viruses, bacteria and fungi, suggesting that this gene represents a crucial node in the crosstalk of stress signaling cascades through the activation of multiple components [85]. To dissect the role of *OsMYB4* in rice, Park and co-workers [86] generated *OsMYB4*-overexpressing rice plants, which displayed a higher peroxidase and total antioxidant activities than wild type plants, as well as a higher germination rate and reduced membrane injuries when taken at above-lethal low temperature (10 °C). A transcriptomic analysis showed that *OsMYB4* controls a large and complex transcriptional network associated with diverse cellular processes, primarily defense and rescue, metabolism and development. The cold-mediated *OsMYB4* transcriptome included genes encoding stress-related proteins and proteins associated with cellular redox homeostasis and detoxification, cellular communication and signal transduction, including TFs belonging to at least 17 different families. This is in accordance with the highly hierarchical nature of *OsMYB4* and suggests that the OsMYB4-mediated regulation of downstream genes can be either direct or mediated by the action of other TFs. The cold-independent *OsMYB4* transcriptome displayed a lower representation of stress-related genes, whereas a large number of genes in this group belong to other categories such as growth, development and morphogenesis, transport mechanism and facilitation, protein biosynthesis, and metabolism. This is probably due to the fact that supra-optimal overexpression of *OsMYB4* affects additional sets of genes that are not directly involved in stress response mechanisms [86]. Furthermore, *OsMYB4* has been ectopically expressed in several species and it is able to improve the tolerance/resistance to various stresses in both monocots and dicots via the activation of different metabolic pathways, such as the chorismate and phenylpropanoid pathways (tomato, apple, *Osteospermum ecklonis* (DC.) Norl., tobacco, *Salvia sclarea* L., *Hordeum vulgare* L. and potato) [87,88,89,90,91,92]. These data suggested that *OsMYB4* is a crucial component of a stress–signaling network which is conserved among species. For this reason, a recent study was conducted to identify the *OsMYB4* gene family in both monocot and dicot species [82]. In particular, OsMYB4 belongs to a small rice subfamily that contains three members (OsMYB4, LOC_Os02g41510 and OsMYB8/LOC_Os10g33810); three subfamilies corresponding to these three TFs were also identified in other monocot species, such as wheat and maize. An expression analysis and an *in silico* promoter analysis confirmed that rice, wheat and *Arabidopsis OsMYB4*-like genes are involved in the response to similar environmental stimuli. Moreover, transient transformation assays demonstrated that OsMYB4 is able to repress the activity of its own promoter and of the promoter of its putative paralogue *Os02g41510*. A compensatory mechanism of auto-regulation is consistent with the well-known complexity of the OsMYB4-activated pathway and suggests that an *in vivo* “feedback control” mechanism may regulate the expression of other *OsMYB4*-like genes [82].

The wheat *TaPIMP1* gene encodes a R2R3-type MYB TF, closely related to *AtMYB108* and *AtMYB2*. It is involved in the tolerance response to both abiotic and biotic stresses. Its nuclear localization and its ability to bind MYB-binding sites have been demonstrated [93,94]. A first study indicated that the *TaPIMP1* transcript level was significantly up-regulated by inoculation with the fungal pathogen *Bipolaris sorokiniana* and by dehydration treatment [93]. Interestingly, the transcript induction of *TaPIMP1* after inoculation depended on the resistant response of the genotype: in a resistant wheat genotype its expression was induced more rapidly and at higher levels compared to a susceptible one, suggesting a putative role of this gene in wheat defense response to certain pathogens. Transgenic tobacco plants expressing *TaPIMP1* showed higher levels of PAL and SOD activities and an enhanced resistance to *Ralstonia solanacearum* infection compared to untransformed plants, and *TaPIMP1* transcript levels were associated to the enhanced level of resistance. Moreover, ectopic expression of *TaPIMP1* in tobacco also resulted in improved tolerance to drought, salt, and oxidative stresses compared to wild type plants. These data suggested that *TaPIMP1* is able to improve tolerance/resistance to abiotic and biotic stresses, probably through an improved ROS scavenging activity [93]. More recently, an in-depth *TaPIMP1* functional characterization using wheat lines overexpressing or underexpressing this gene was reported [94]. *TaPIMP1*-overexpressing plants displayed an enhanced resistance to the hemibiotrophic fungal pathogen *B. sorokiniana*, showing an inhibition of mycelial growth, and an improved tolerance to drought stress, due to earlier closure of stomata, increased proline content and reduced water loss. As observed in tobacco transgenic lines, the degree of resistance/tolerance was correlated with *TaPIMP1* expression levels. Microarray data showed that the overexpression of *TaPIMP1* in wheat induced 112 transcript sets, which are involved in defense, stress response and signal transduction. In particular, the transcript of some defense- and stress-related genes up-regulated by *TaPIMP1*, including *RD22*, *PR1a*, *TLP4*, *GST22*, *GLP4*, *dehydrin 6*, *ABAI*, *PR2*, and *PAL5*, were also induced by both *B. sorokiniana* infection and drought stress, suggesting that the wheat response to these two environmental constraints were partially overlapping. A further expression analysis indicated that *TaPIMP1* as well as these defense- and stress-related genes activated by *TaPIMP1* were induced by ABA and SA treatments to a greater and a lesser extent in the *TaPIMP1*-overexpressing and repressing plants than in the wild type plants, respectively. These data clearly revealed that TaPIMP1 is a positive mediator in wheat responses to *B. sorokiniana* and drought stress and suggested that it may regulate defense- and stress-related genes in the ABA and SA signaling pathways [94].

A further gene involved in the response to both fungal pathogens and abiotic stresses is *SpMYB*, which encodes a R2R3-type MYB protein and has been isolated from a wild species of tomato (*Solanum pimpinellifolium* L.) plants infected by *Phytophthora infestans* [95]. Its expression was significantly induced by inoculation with *P. infestans* and reached the highest peak at 6 h after the inoculation. Its ectopic expression in tobacco conferred tolerance to salt and drought stresses. Transgenic lines showed a more developed root system and a higher fresh biomass compared to wild type plants under salt or drought stresses, as well as a minor growth inhibition. Moreover, ectopic expression of *SpMYB* conferred increased resistance to *Alternaria alternata* infection. Indeed, seven days after inoculation, tobacco transgenic lines displayed lower disease index and MDA content, a higher activity of SOD, POD and PAL and a better photosynthetic performance in leaves than wild type tobacco plants, as well as a higher expression level of *NtPR1* and *NtPR2* genes. These results suggested that *SpMYB* may confer plant resistance in response to *A. alternata* infection through the activation of different defense systems, such as enhanced antioxidant enzymes activities or the activation of *PR* genes [95].

## 5. Post-Transcriptional Control of Some *MYB* Genes Involved in Drought Stress Response

TF activities are finely regulated at various steps in diverse cellular signaling networks for optimal growth and survival under different growth conditions [150,151]. Well-established molecular and biochemical mechanisms underlying regulation of TF activities include gene transcriptional regulation, post-transcriptional regulation of RNA metabolism, protein translation, post-translational modifications, and controlled protein turnover. In particular, the existence of different mechanisms of post-transcriptional control of mRNA metabolism is a key molecular scheme that modulates the TF activities in plant responses to environmental cues [152].

Among them, alternative splicing provides proteome diversity and, thus, expands the repertoire of gene/protein activities in response to developmental and environmental cues [153,154]. Several MYB TFs are regulated through mechanisms involving alternative splicing [12,155,156,157]. Recently, the *QsMYB1* gene identified in oak (*Quercus suber* L.) has been associated to the drought response and to alternative splicing mechanisms [96,97]. *QsMYB1* codes for an R2R3-type MYB TF and its expression is putatively mediated by an alternative splicing mechanism that originates two different transcripts (*QsMYB1.1* and *QsMYB1.2*), differing in the 5′-untranslated region, with the *QsMYB1.2* variant retaining the first intron. Expression analysis of *QsMYB1* transcripts showed that both transcripts were differently regulated in cork tissues and organs and that their amount was differently regulated by abiotic stress conditions [96,97]. In particular, increasing temperatures led to a gradual down-regulation of *QsMYB1* splicing variants with a stronger effect on *QsMYB1.1* abundance, whereas under drought condition *QsMYB1* transcripts were transiently up-regulated, and *QsMYB1.2* variant showed the highest drought-dependent induction. Both these two abiotic stresses followed by recovery triggered changes on the expression profile of the two *QsMYB1* splicing variants indicating that the *QsMYB1* TF is modulated at the post-transcriptional level by heat and drought stresses, which affect mainly the regulation of the spliced transcript *QsMYB1.1* and of the un-spliced variant *QsMYB1.2*, respectively [97].

Another important mechanism of post-transcriptional regulation of RNA involved the microRNAs (miRNAs), a class of small, non-coding, endogenous RNAs, that play negative regulatory roles in gene expression. In plants, miRNAs are involved in several processes, including development, hormone regulation, nutrient homeostasis, and stress response [158,159,160,161]. Recently, several studies have shown a pivotal role of miRNAs in drought tolerance via control of the expression of drought-responsive genes, most of them encoding TFs [160,161]. The miR159 family members regulate the expression of *GAMYB*-like genes [162,163,164,165]. *GAMYB*-like genes are present in many plant species and are phylogenetically similar to the *HvGAMYB* gene isolated from barley, which is a positive regulator of the GA signal transduction pathway in the barley aleurone [166]. In *Arabidopsis*, there is a small family of *GAMYB*-like genes, consisting of 5 *MYB* genes (*AtMYB33*, *AtMYB65*, *AtMYB97*, *AtMYB101* and *AtMYB120*); among them, *AtMYB33*, *AtMYB65*, and *AtMYB101* may mediate GA signaling in growth and flowering responses [98]. *AtMYB33* was previously found to be a miR159 target during leaf morphogenesis [99,100]. Moreover, *AtMYB101* and *AtMYB33* act as positive regulators of ABA signaling during germination and miR159 is involved in the post-transcriptional control of their expression to desensitize hormone signaling during seedling stress responses [101]. In germinating seeds, ABA induced the accumulation of miR159 in association with the seed specific TF ABI3. In turn, miR159 mediated the cleavage of *AtMYB101* and *AtMYB33* transcripts and its overexpression suppressed *AtMYB101* and *AtMYB33* transcript levels, leading to an ABA hyposensitive response. These data suggested that ABA-induced accumulation of miR159 is a homeostatic mechanism to desensitize ABA signaling via *AtMYB101* and *AtMYB33* transcript degradation [101]. Moreover, miR159 levels are modulated by GA during anther development [100]. Reyes and Chua [101] proposed a model in which ABA and GA signaling pathways trigger miR159 accumulation through a transcriptional control, with the ABA- or GA-mediated accumulation of miR159 depending on developmental context: miR159 expression is regulated by ABA during seed development and by GA during flower development.

A recent study identified three novel miR159 family members (stu-miR159a, stu-miR159b and stu-miR159c) in potato and their targeted *GAMyb*-like genes (*StGAMyb-like1*, *StGAMyb-like2.1* and *StGAMyb-like2.2*), putatively involved in drought stress response [102]. The expression level of the three stu-miR159 members significantly decreased after 25 days of drought stress treatment, whereas the expression of their targeted *GAMyb*-like genes greatly increased, suggesting that stu-miR159s negatively regulated the expression of potato *GAMyb*-like genes. These results give a new insight into the role of *GAMyb*-like genes, which seem to be involved not only in ABA response during germination, but also in the response to water stress.

## 6. Conclusions and Future Perspectives

Understanding the regulatory gene network responsive to drought can help both researchers and breeders in manipulating plants to improve stress tolerance and productivity. Plant TFs play a major role in the regulation of drought response [6]. Among them, numerous MYB TFs involved in the regulation of drought tolerance have been identified, particularly in the model species *Arabidopsis thaliana*, as described in this review. Several of these MYB TFs can be considered useful targets of biotechnological manipulation to improve drought tolerance, through their overexpression or silencing, and examples of this approach have been described throughout the text. Moreover, marker assisted selection (MAS) programs, turned to improve drought tolerance, could exploit the potentiality of MYB TFs. Until now, the use of MYB TFs in MAS programs related to drought tolerance has been very rare. An interesting example concerns the development of a molecular marker on the *TaMYB2* gene in wheat [103]. *TaMYB2* codes for an *AtMYB2* ortholog and its expression is up-regulated in response to different stresses such as dehydration, salinity, cold and to ABA treatment. The positive transcriptional response to dehydration is particularly evident in two dehydration tolerant cultivars compared to two sensitive ones. Re-sequencing of the *TaMYB2* gene in these four wheat cultivars revealed the presence of a synonymous SNP at the 458^th^ bp position (A/G transition): the tolerant cultivars present an “A”, while the sensitive cultivars a “G” allele. Based upon this sequence analysis, an allele-specific marker was developed and validated through the association of this molecular marker with the tolerance trait on additional 16 and eight dehydration-tolerant and -sensitive wheat cultivars, respectively. Since tolerant cultivars showed increased *TaMYB2* expression level compared to the sensitive ones, the authors speculate that it can most likely be due to any post-transcriptional gene modification(s) or to any deficiency in the stress responsive *cis*-regulatory element(s) present in the promoter regions of the sensitive cultivars [103].

Although *MYBs* are good candidate genes to be used for improving drought tolerance in crops, so far the information acquired on *MYB* function has scarcely been used for crop breeding. One of the major limits is that the majority of the functional studies have been performed under laboratory conditions and the transfer of the results to the field is very difficult, since the field environment is very different from controlled laboratory conditions. Moreover, different stresses simultaneously occur in the field, and recent studies revealed that the response to multiple environmental cues cannot be simply extrapolated from the sum of the effects of the different stresses singularly applied [106]. Then, it is very important to study the combined effect of different stresses. In this context, it is noteworthy that many *MYB* genes, which are involved in the regulation of drought response, are also implicated in the response to other stresses, as reported in Section 4 “Crosstalk among Different Stress Responses”. This aspect makes the MYB TFs good candidates to improve stress tolerance and productivity in plants, providing valuable starting points to develop crop protection against multiple stresses.
